# Amino Acid Sensor Kinase Gcn2 Is Required for Conidiation, Secondary Metabolism, and Cell Wall Integrity in the Taxol-Producer *Pestalotiopsis microspora*

**DOI:** 10.3389/fmicb.2017.01879

**Published:** 2017-09-27

**Authors:** Dan Wang, Oren Akhberdi, Xiaoran Hao, Xi Yu, Longfei Chen, Yanjie Liu, Xudong Zhu

**Affiliations:** ^1^National Key Program of Microbiology and Department of Microbiology, College of Life Sciences, Nankai University, Tianjin, China; ^2^National Experimental Teaching Demonstrating Center, School of Life Sciences, Beijing Normal University, Beijing, China; ^3^Beijing Key Laboratory of Genetic Engineering Drug and Biotechnology, Institute of Biochemistry and Molecular Biology, School of Life Sciences, Beijing Normal University, Beijing, China

**Keywords:** Gcn2, secondary metabolism, pestalotiollide B, conidiation, *Pestalotiopsis microspora*

## Abstract

The canonical Gcn2/Cpc1 kinase in fungi coordinates the expression of target genes in response to amino acid starvation. To investigate its possible role in secondary metabolism, we characterized a *gcn2* homolog in the taxol-producing fungus *Pestalotiopsis microspora*. Deletion of the gene led to severe physiological defects under amino acid starvation, suggesting a conserved function of *gcn2* in amino acid sensing. The mutant strain *Δgcn2* displayed retardation in vegetative growth. It generated dramatically fewer conidia, suggesting a connection between amino acid metabolism and conidiation in this fungus. Importantly, disruption of the gene altered the production of secondary metabolites by HPLC profiling. For instance, under amino acid starvation, the deletion strain *Δgcn2* barely produced secondary metabolites including the known natural product pestalotiollide B. Even more, we showed that *gcn2* played critical roles in the tolerance to several stress conditions. *Δgcn2* exhibited a hypersensitivity to Calcofluor white and Congo red, implying a role of Gcn2 in maintaining the integrity of the cell wall. This study suggests that Gcn2 kinase is an important global regulator in the growth and development of filamentous fungi and will provide knowledge for the manipulation of secondary metabolism in *P*. *microspora*.

## Introduction

Amino acids are the fundamental nutrients and building blocks of the cell. Eukaryotic organisms have evolved mechanisms for monitoring amino acid availability to coordinate metabolism and development. One of these is the protein kinase Gcn2-mediated amino acid sensing system, which was first identified in *Saccharomyces cerevisiae* and is known as the ‘general amino acid control’. Conserved regulation known as the ‘cross-pathway control’ (Cpc) is also found in filamentous fungi. The action of Gcn2, which occurs at the translation initiation step, has been well illustrated in yeast. In amino acid starvation, uncharged tRNA accumulates in the cell, which activates the Gcn2 kinase catalytic activity. The direct target protein of the activated Gcn2 is the translation initiation factor 2 (eIF2α), which is phosphorylated at Ser-51 by Gcn2 (Baird and Wek, 2012). This causes a global inhibition of translation, but simultaneously promotes translation of the set of mRNAs for amino acid biosynthesis and transport (Natarajan et al., 2001; Kubota et al., 2003).

The Gcn2 kinase consists of multiple functional domains, some of which can undergo self-inhibition/activation by interaction with each other. The catalytic kinase domain (KD) is initially inert and converts to an active conformation through interactions with four other domains (Qiu et al., 2002). A unique structural feature of Gcn2 is a tRNA-binding domain located next to the C-terminal KD, which is homologous to the tRNA-binding domain of histidyl-tRNA synthetase (HisRS). This domain plays a sensor role by binding uncharged histidyl-tRNA to subsequently activate Gcn2 in amino acid-depleted conditions (Wek et al., 1995). The extreme C-terminal domain (CTD) plays either a positive or negative role in kinase regulation: dimerization and ribosome binding activate the kinase activity of Gcn2, whereas autoinhibition of the KD leads to a negative effect on the kinase activity (Zhu and Wek, 1998).

Apart from regulating amino acid metabolism, Gcn2/Cpc1 kinases are involved in controlling developmental processes in yeasts and filamentous fungi. In the yeast *S. cerevisiae*, activation of this signal causes the cell to favor adherence to a surface (Braus et al., 2003). The GCN pathway in the pathogenic yeast *Candida albicans* affects a dimorphic shift that is critical for pathogenicity (Tournu et al., 2005). Similarly, the counterpart of Gcn2 in the plant vascular pathogen *Verticillium longisporum*, Cpc1, has been demonstrated to be required for pathogenicity (Timpner et al., 2013). The Cpc pathway in *Aspergillus nidulans* negatively regulates the sexual developmental stage, i.e., its activation impairs the formation of fruiting body cleistothecia (Hoffmann et al., 2001). Despite the above significant findings, knowledge about the function of the Gcn2/Cpc kinases in filamentous fungi is limited.

In this study, we identified a homolog of *gcn2*/*cpc1* in the genome of NK17, and designated as *gcn2*. Via loss-of-function manipulation, we created a targeted disruption of *gcn2* strain in this fungus. We demonstrate that it is involved in the response to amino acid starvation. *gcn2* is critical for asexual sporulation and secondary metabolite biosynthesis, suggesting Gcn/Cpc pathway has variable functions in filamentous fungi.

## Materials and Methods

### Strains and Culture Conditions

*Pestalotiopsis microspora* NK17 was previously isolated by our laboratory as a taxol-producing strain (Bi et al., 2011). Its uracil auxotrophic strain, *Δura3-NK17*, was created and used as the recipient strain in this study. Fungal strains were grown or maintained in potato lactose broth (PLB, using 2% lactose instead of glucose) at 25–28°C, with shaking at 180 rpm, or on 2% (w/v) agar (PLA) at 25–28°C. If needed, the fungal strains were also cultivated on glucose (2%) asparagine salt medium (2% asparagine, 0.3% KH_2_PO_4_, pH 5.2; Asn) as a minimal medium. All bacterial strains were grown in LB medium with appropriate antibiotics added when needed, at 28°C (for *Agrobacterium tumefaciens*) or 37°C (for *Escherichia coli*), with shaking at 180 rpm. Inducing medium (IM) and Yeast Nitrogen Base (YNB) medium were used for the transformation of NK17. The histidine analog 3-Aminotriazole (3AT) was used at the concentrations specified.

### *P. microspora gcn2* Mutant Strain Construction

All primers used in this study are listed in **Table 1**. The deletion vector pOSCAR-Gcn2 was constructed by the OSCAR protocol described previously (Paz et al., 2011). The pA-Hyg-OSCAR marker vector was used, with *pm-ura3* replacing *Hph* as the selective marker. The upstream and downstream homologous arm PCR fragments were obtained and gel purified separately using the AxyPrep DNA Gel Extraction Kit (Axygen, Union City, CA, United States). The deletion construct was set up using BP Clonase II enzyme (Invitrogen, Carlsbad, CA, United States). Then, the reaction mixture was transformed into *E. coli* DH5α. Bacterial colonies were obtained on LB plates with 100 μg mL^-1^ spectinomycin, following overnight incubation at 37°C. Two pairs of primers, Gcn2-up (F)/Ura3 (R) and Ura3 (F)/Gcn2-down (R), were used to verify the deletion construct, pOSCAR-Gcn2.

**Table 1:** Primers used in this study.

Primer	Sequence (5′→3′)
Gcn2-up(F)	GGGGACAGCTTTCTTGTACAAAGTGGAA ACGCACTGGAACAGCATG
Gcn2-up(R)	GGGGACTGCTTTTTTGTACAAACTTGT GAAGCGAGTACGAACCCT
Gcn2-down(F)	GGGGACAACTTTGTATAGAAAAGTTGTT TTCGCTTGACCGTAAATCCG
Gcn2-down(R)	GGGGACAACTTTGTATAATAAAGTTGT GGGCAAGCCGCCGTCACTAT
Gcn2(F)	TTTGTGAGATTTCCCGACTT
Gcn2(R)	AACGCTCGGAACCAGCCTTT
Gcn2-PRO(F)	GGGGACAGCTTTCTTGTACAAAGTGGAAGATTGAGTAGATAAGCGGAG
Gcn2-TER(R)	GGGGACAACTTTGTATAATAAAGTTGT TCAGATTTATCAGAAAGGGA
Ura3(F)	CGAGGTCGACATAACTTCGT
Ura3(R)	ACGAAGTTATTTCACTGGCA
Hyg(F)	GCCCTTCCTCCCTTTATT
Hyg(R)	TGTTGGCGACCTCGTATT
ACTIN1	GTCGCTGCCCTCGTTATC
ACTIN2	CGAGAATGGAACCACCGA
ACTIN3	CCCAAGTCCAACCGTGAGAA
ACTIN4	GGAGTCGAGCACGATACCGG
Gcn4-RT1	GTGGTCACGCTCAGCCTCAA
Gcn4-RT2	GTCTCTGGCGTCAATGCTCG
PKS1-RT1	GCCATAGGGAATAACGAGAA
PKS1-RT2	AGAGACAGAGACCAAAGCCC
MAPK-RT1	CCTTTCCTACTGTCGGCACG
MAPK-RT2	GACCGCTTCCAGCAGAGATG
P450-RT1	GTGCTGCTTGAACGAAATGC
P450-RT2	CCGAGCCTGTAGTGGACGAA

Disruption of *gcn2* in *P. microspora* was achieved through *A. tumefaciens*-mediated genetic transformation. pOSCAR-Gcn2 was transformed into *A. tumefaciens* LBA4404 using a protocol described previously (Chen et al., 2015; Yu et al., 2015b). Then, *A. tumefaciens* LBA4404 containing pOSCAR-Gcn2 was cocultured with 10^7^ conidia from *P. microspora* strain *Δura3-NK17* at 28°C on a nitrocellulose filter that was spread on an IM plate supplemented with 50 mg L^-1^ uracil and 40 mg L^-1^ acetosyringone (Sigma, St. Louis, MO, United States). After induction for 2 days, the filter was transferred onto an YNB plate supplemented with cefotaxime (100 mg L^-1^) followed by incubation for 2 days at 28°C for sporulation. Individual fungal transformants were obtained through single-spore isolation.

### Complementation of *gcn2* and Southern Blotting

The complementation plasmid pOSCAR-Gcn2-C was constructed using the BP Clonase reaction and pA-Hyg-OSCAR (Invitrogen, Carlsbad, CA, United States) was used, which contains a fungal marker *Hph*. The complementation strain was created by introducing a 6.6-kb fragment containing the wild-type (WT) copy of *gcn2* into *Δgcn2*. Genomic DNA was used as template in PCR.

Total DNA from each strain was extracted from mycelium grown in 200 mL PLB for 4 days, as described by Hao et al. (2012). PCR amplification and Southern blotting were used to characterize the *Δgcn2* strain and the complementation strain *Δgcn2*::GCN2. For PCR validation, two pairs of primers were used, Gcn2 (F)/Ura3 (R) and Gcn2 (R)/Ura3 (F) (**Table 1**). Southern blotting was conducted as described previously (Zhang et al., 2015) to confirm insertion/deletion of *gcn2* in the various strains.

### RNA Preparation, Reverse-Transcription PCR, and Quantitative Real-Time PCR

Total RNA was prepared from lyophilized mycelia using the TRIzol Kit (Invitrogen, Carlsbad, CA, United States). To remove possible contaminant DNA, the RNA samples were then treated with RNase-free DNase (Takara, China). Reverse-transcription PCR (RT-PCR) and quantitative real-time PCR (qRT-PCR) were performed as described previously (Zhang et al., 2015, 2016).

To examine the expression of *gcn2*, *gcn2* mRNA was amplified by RT-PCR. Total RNA was isolated from fresh mycelia grown in PLB, at 28°C, for 2, 3, 4, 6, and 8 days, respectively. Double-stranded cDNA was synthesized using a M-MLV RTase cDNA Synthesis Kit (Takara, China), followed by RT-PCR for 28 cycles as determined beforehand. As a control, the mRNA of the actin-encoding gene *ACT1* of NK17 was amplified in parallel. qRT-PCR was performed on a Mastercycler PCR machine (Eppendorf, Germany). Each reaction (20 μL) was carried out with SYBR Green I PCR Master Mix (Roche China, Shanghai). Reactions were set up in duplicate. Controls without addition of template were included for each primer pair. PCR cycling parameters were: denaturation at 94°C for 10 min, followed by 40 cycles of denaturation at 94°C for 15 s, annealing at 59°C for 30 s, and extension at 72°C for 32 s. The qRT-PCR data were analyzed using the 2^-ΔΔC_T_^ relative quantification method. The actin housekeeping gene mRNA served as the internal reference. The amplification efficiencies of the target and reference genes were compared at different template concentrations.

### Quantification of Conidia Production and Dry Weight of Mycelia

Conidia were harvested from cultures inoculated on PLA at 28°C for 8 days. The plates were washed twice with sterile distilled water and the concentration of conidia suspension in sterile distilled water was determined by hemocytometry. Total mycelium was isolated from cultures inoculated in PLB, at 28°C, for 2, 3, 4, 5, 6, 8, and 10 days, respectively. Fresh mycelium was isolated by vacuum pump and dry mycelium was obtained by vacuum freeze–drying. The weight of the dry mycelium was determined.

### Phenotype Observation and Secondary Metabolite Profiling of *Δgcn2*

Equal numbers of conidia (∼5 × 10^4^) from NK17, *Δgcn2* and the complementation strain *Δgcn2*::GCN2 were inoculated into PLB, or onto PLA or Asn (2% glucose) plates, respectively, for characterizing the variation in phenotype. To analyze the secondary metabolites, equal numbers of conidia were cultured in 200 mL PLB, 28°C, with shaking at 180 rpm, for 182 h. Then the culture was extracted with an equal volume of dichloromethane as described previously (Yu et al., 2015a). HPLC profiling was performed by following procedures used previously.

### Drug and Stress Sensitivity Assay

For drug and stress sensitivity assays, 5 × 10^4^ conidia from NK17, *Δgcn2*, and the complementation strain *Δgcn2*::GCN2 were incubated on PLA plates separately supplemented with 0.02% Congo red, 0.5 M KCl, 1 M NaCl, 2 mM sorbitol, 2 mM NaNO_2_, 100 μg/mL G418, 4 μg/mL nystatin and 50 ng/mL calcofluor white. All cultures were incubated at 28°C for 7 days.

## Results

### Characterization of the Amino Acid Sensor *gcn2* in *P. microspora*

**FIGURE 1:**
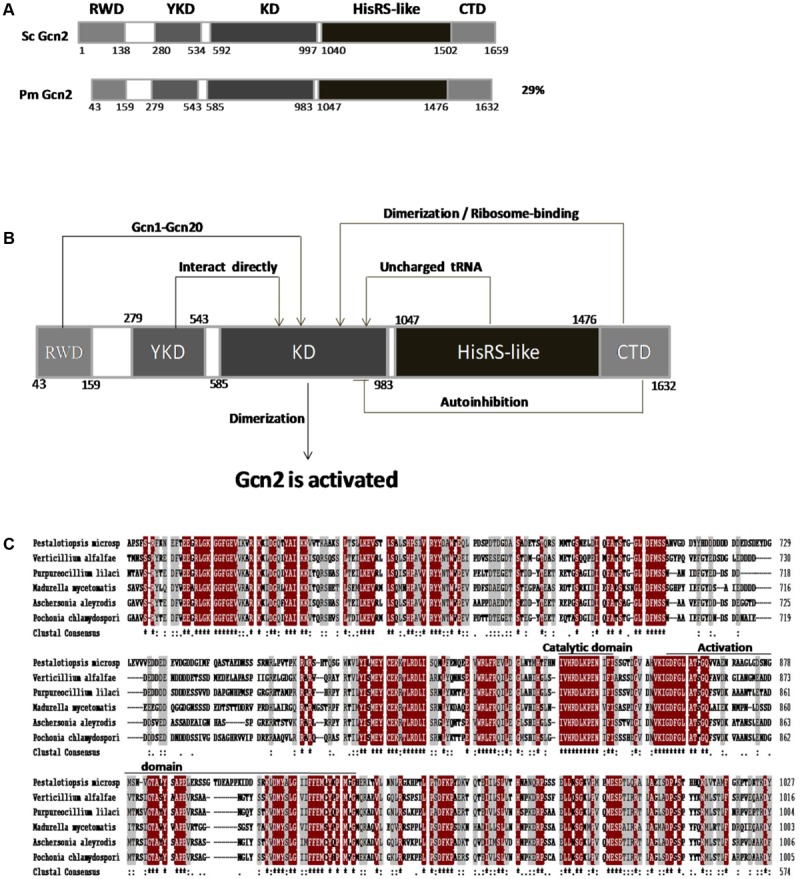
An amino acid sensor homologous to Gcn2 present in *Pestalotiopsis microspora* NK17, and the domain interactions in Gcn2. **(A)** Comparison of *P. microspora* Gcn2 to the homolog *S. cerevisiae* Gcn2 (NP_010569.3); numbers indicate the length and location of the domains in the proteins, including the RWD domain, pseudokinase domain (YKD), kinase domain (KD), HisRS-like domain, and C-terminal domain (CTD). The overall percentage of amino acid sequence identity with *Sc*Gcn2 is shown on the right. **(B)** The domain interactions and activation of Gcn2. Arrows indicate positive domain interactions with the KD to control the kinase activity. Autoinhibition of the CTD is depicted with a plain short line between the CTD and KD. **(C)** CLUSTAL W analysis of the KDs of Gcn2 kinases from *P. microspora*, *Verticillium alfalfae* (XP_003008521.1), *Purpureocillium lilacinum* (XP_018176632.1), *Madurella mycetomatis* (KXX78754.1), *Aschersonia aleyrodis* (KZZ98333.1), and *Pochonia chlamydosporia* (XP_018142183.1). Identical residues are highlighted in dark red and similar residues are shown in light gray. Asterisks represent consensus residues conserved in the kinases.

The genome of *P. microspora* NK17 has been sequenced (unpublished data). When BLAST searching with the amino acid sequence of *GCN2* from *S. cerevisiae* as the query, only one protein similar to *S. cerevisiae* GCN2 was found in *P. microspora* NK17 (29% identity) (**Figure 1A**). The open reading frame (ORF) of *gcn2* in *P. microspora* is 5133 bp long, encoding a peptide of 1632 amino acids (GenBank accession no. KY703869). Structural analysis of Gcn2 suggested that this protein contains a RWD domain (residues 43–159), a pseudokinase domain (YKD) (residues 279–543), a KD (residues 585–983), a HisRS-like domain (residues 1047–1476), and a CTD (1476–1632 residues) (**Figures 1A,B**). Domains involved in the activation of the kinase activity of Gcn2 are also shown in **Figure 1B**. Amino acid sequence alignment of the KD indicated that it shares high identity with the KD of Gcn/Cpc homologs in other filamentous fungi (**Figure 1C**). In addition, a highly conserved motif in the catalytic domain is present in this kinase (**Figure 1C**). The high similarity among these sequences suggests a conserved function of Gcn2 in *P*. *microspora*.

**FIGURE 2:**
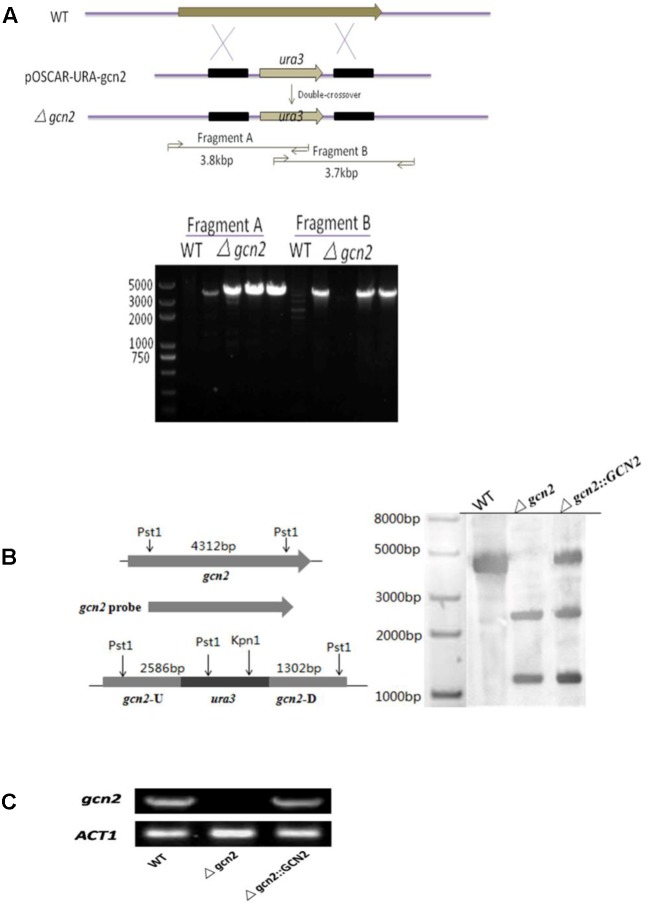
Deletion of *gcn2* from *P. microspora* NK17. **(A)** The disruption cassette was carried on the plasmid pOSCAR-URA-Gcn2 and *gcn2* was replaced by a NK17 *ura3* marker through homologous recombination. Two fragments, of 3.8 and 3.7 kb, were amplified from *Δgcn2*, while no band was seen for NK17. The primers used, Gcn2 (F), Gcn2 (R), Ura3 (F), and Ura3 (R), are listed in **Table 1**. **(B)** Southern blotting to confirm the deletion of *gcn2*. Genomic DNAs from NK17, *Δgcn2* and the complementation strain *Δgcn2*::GCN2 were digested with *Kpn*I and *Pst*I. The probe used for Southern blotting was amplified by primers Gcn2-up (F) and Gcn2-down (R). Two bands on the membrane, at 2.6 and 1.3 kb, were obtained for *Δgcn2*, while in wild-type (WT) NK17, one band of 4.3 kb was observed. In the complementation strain *Δgcn2*::GCN2, there were three bands (2.6, 1.3,and ∼4.8 kb). **(C)** Transcription of *gcn2* was detected in NK17 and the complementation strain *Δgcn2*::GCN2, but no transcription of *gcn2* was identified in *Δgcn2*.

To investigate the function of *gcn2*, we replaced the genomic copy with a selection marker (the NK17 *ura3* gene) via homologous recombination (**Figure 2A**). We identified disruptants by PCR screening in which two pairs of primers were used to generate bands of 3.8 kb (fragment A) and 3.7 kb (Fragment B) (**Figure 2A**). One of the candidate disruptants, designated strain *Δgcn2*, was picked for Southern blotting analysis, which confirmed the correct position of a single copy of the marker insertion (**Figure 2B**). A fragment carrying a 6.6-kb fragment of the WT *gcn2* was reintroduced into strain *Δgcn2* to restore the deficient phenotype of *Δgcn2*. One of the complemented transformants was single–conidium purified and served as control in Southern blots in parallel with the WT. Loss of *gcn2* mRNA from *Δgcn2* was confirmed by RT-PCR, but this mRNA was detected in WT NK17 and the complementation strain *Δgcn2*::GCN2 (**Figure 2C**).

### *gcn2* Is Required for Conidiation and Vegetative Growth under Amino Acid Starvation

**FIGURE 3:**
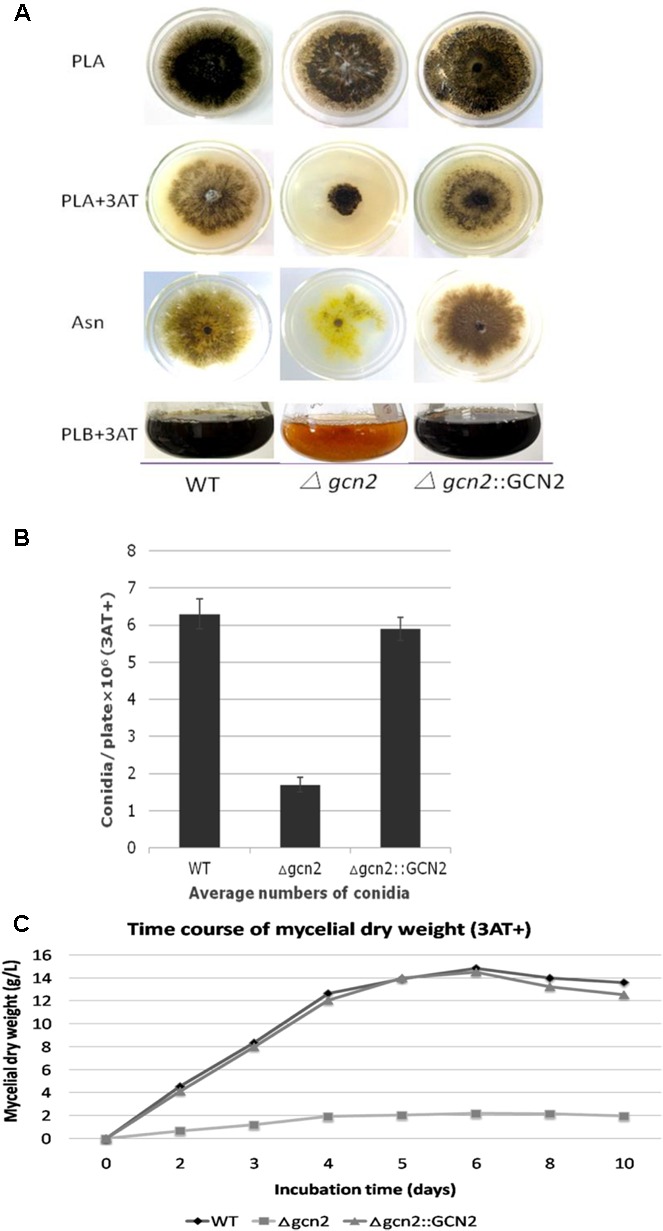
Phenotype characterization of strain *Δgcn2* during amino acid starvation. **(A)** A similar growth rate of the WT, *Δgcn2* and the complementation strain *Δgcn2*::GCN2 was observed on PLA (the top row of plates). On PLA + 3AT (5 mM), growth of *Δgcn2* was seriously delayed (the second row of plates from the top), compared with PLA alone (top row). Compared to the WT NK17 and the complementation strain *Δgcn2*::GCN2, less conidiation was observed for *Δgcn2* on PLA (the top row) and on Asn agar (the third row). Defective conidiation (less pigmentation, indicating less conidia) was also observed in PLB (the bottom row). **(B)** The number of conidia produced by NK17, *Δgcn2* and *Δgcn2*::GCN2 was determined as 6.22 ± 0.45 × 10^6^ (*p* < 0.01), 1.79 ± 0.24 × 10^6^ (*p* < 0.01) and 5.84 ± 0.28 × 10^6^ (*p* < 0.01), per plate, respectively. Triplicate PLA + 3AT (5 mM) plates for each strain were incubated at 28°C for 168 h and used for quantification. Error bars represent standard deviations. **(C)** A growth curve based on the quantity of dry-weight of mycelia for NK17, *Δgcn2* and the complementation strain *Δgcn2*::GCN2 in the presence of 3AT (5 mM) in PLB at the indicated time points. Dry weight was obtained as the mean value of three parallel cultures. Each strain was incubated at 28°C for 168 h.

As yeast *gcn2* is involved in response to amino acid starvation, we first tested whether *P. microspora* NK17 *gcn2* has a similar role. We cultivated the fungal strains NK17, *Δgcn2*, and the complementation strain *Δgcn2*::GCN2 on different agar including PLA, PLA with 3AT (5 mM), Asn plates, and in PLB containing 3AT (5 mM), as indicated (**Figure 3A**). The three strains seemed to have similar growth rates on the complete medium PLA (**Figure 3A**, top row). However, we found that *Δgcn2* was sensitive to 3AT and suffered severe growth retardation. A growth curve (based on dry weight of mycelia) was constructed for *Δgcn2* and the complementation strain *Δgcn2*::GCN2 in the presence of 3AT (5 mM) in PLB (**Figure 3C**). Dry weight of mycelia was measured in triplicate. We observed that the growth of strain *Δgcn2* was substantially impaired.

We noticed at the same time that pigmentation of the mycelium of *Δgcn2* seemed to be affected on PLA and Asn (**Figure 3A**). As melanin biosynthesis is associated with the formation of conidia in NK17 (Yu et al., 2015a), we checked the conidiation of strain *Δgcn2*. Disruption of *gcn2* led to poor conidiation on PLA or Asn (**Figure 3B**). On Asn plates (where asparagine served as the sole nitrogen source), the mutant strain *Δgcn2* produced an average of 1.79 ± 0.24 × 10^6^ (*p* < 0.01) conidia per plate, whereas NK17 produced about 6.22 ± 0.45 × 10^6^ (*p* < 0.01) conidia per plate. The complementation in strain *Δgcn2*::GCN2 restored the number of conidia to 5.84 ± 0.28 × 10^6^ (*p* < 0.01) conidia per plate. This result suggests that deletion of *gcn2* affected the production of conidia in *P. microspora*. Furthermore, compared with NK17, *Δgcn2* produced very little mycelium (**Figure 3C**). Taken together, *gcn2* plays a critical role in conidiation and vegetative growth in *P. microspora* NK17.

### Sensitivity of *Δgcn2* to External Stress

**FIGURE 4:**
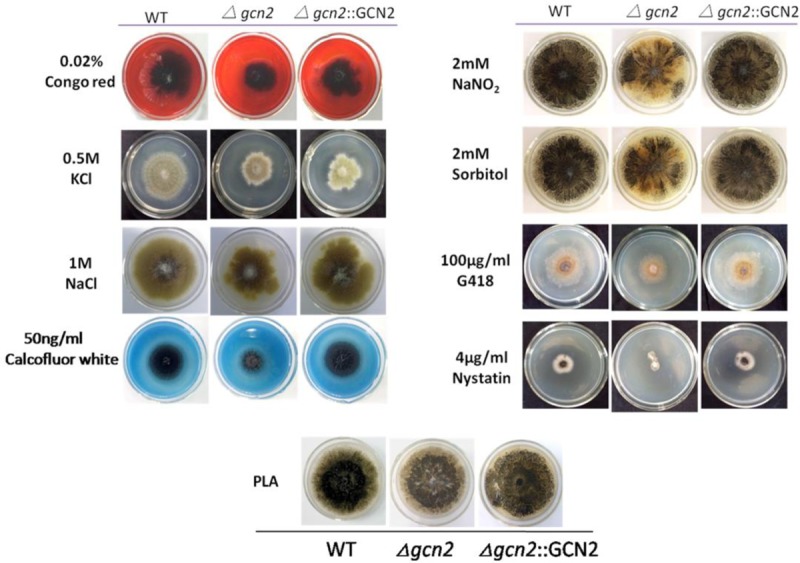
Susceptibility tests for NK17, *Δgcn2*, and the complementation strain *Δgcn2*::GCN2. Stress tolerance to Congo red, KCl, NaCl, sorbitol, calcofluor white, external oxidative stress from NaNO_2_, and susceptibility to G418 and nystatin on PLA. PLA without any supplements served as the control. The layout of strains is indicated at the top of the Figure. Conidiation of *Δgcn2* was discernibly affected by 2 mM NaNO_2_ and sorbitol. *Δgcn2* showed sensitivity to 0.02% Congo red, 50 ng/mL calcofluor white, 0.5 M KCl, 1 M NaCl, 100 μg/mL G418 and 4 μg/mL nystatin.

*S. cerevisiae gcn2* plays a role in stress resistance, including to oxidative stress, saline stress, and antifungal agents. To test the role of NK17 *gcn2* in response to stress conditions, assays were performed in the presence of the following chemical agents at appropriate concentrations: cell wall inhibitors Congo red and calcofluor white; osmotic reagents KCl and NaCl; oxidant NaNO_2_; osmotic stabilizer sorbitol; and the antifungal agents G418 and nystatin. Addition of 0.02% Congo red to PLA plates resulted in slower growth of *Δgcn2* compared with the controls (**Figure 4**, left panels). Moreover, addition of 50 ng/mL calcofluor white led to marked inhibition of the growth of the mutant strain, while the WT and the complementation strain grew well (**Figure 4**, left panels). We suggest that the integrity of the cell wall in NK17 was affected by the deletion of *gcn2.* Treatment with 0.5 M KCl and 1 M NaCl could distinguishably inhibit conidiation and retard the vegetative growth of the mutant strain (**Figure 4**) compared with the control strains (the PLA plates in **Figure 4**). This result indicated that cellular responses to osmotic changes in NK17 require *gcn2.* On plates supplemented with the oxidant 5 mM NaNO_2_, *Δgcn2* obviously produced less conidiation (by an estimate of pigmentation) compared with WT NK17 and the complementation strain, implying a role of *gcn2* in the response to reactive oxygen species. A similar phenotypic outcome in conidiation was observed in the response of *Δgcn2* to 2 mM sorbitol. Lastly, the mutant strain *Δgcn2* exhibited hypersensitivity to 100 μg/mL G418 and 4 μg/mL nystatin (**Figure 4**, right panel).

### Roles of *gcn2* in the Biosynthesis of Secondary Metabolites and Mycelial Pigmentation

**FIGURE 5:**
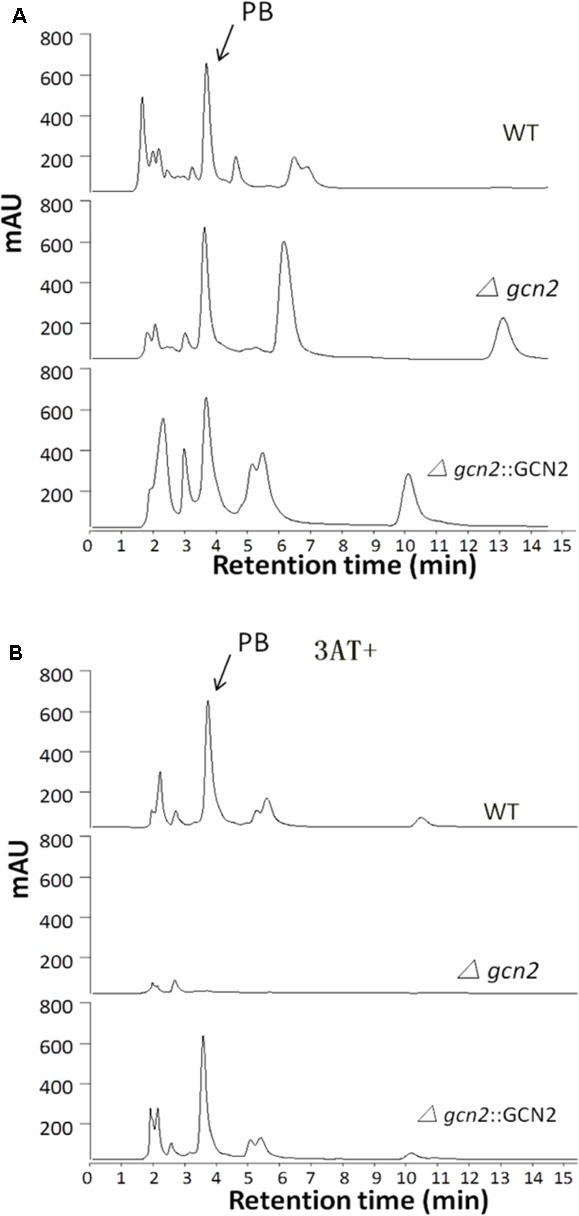
HPLC profiling of secondary metabolite biosynthesis. **(A)** HPLC profiling of the secondary metabolites in *Δgcn2*. Samples were prepared from cultures in PLB. Pestalotiollide B, a characterized product of NK17, is indicated and was produced at a similar level by all three strains. The peak pattern of *Δgcn2* was distinct from that of the wild-type. **(B)** HPLC profiling of the secondary metabolites in *Δgcn2* with 3AT. During amino acid starvation created with 3AT (5 mM), the general production of secondary metabolites in *Δgcn2* showed a dramatic decrease compared to NK17 and the complementation strain *Δgcn2*::GCN2.

Pathway-specific and global transcriptional regulators coordinate the production of secondary metabolites in filamentous fungi (Dufour and Rao, 2011; Rohlfs and Churchill, 2011). To investigate whether Gcn2 was required for controlling the process in NK17, we conducted HPLC profiling for the mutant strain *Δgcn2* (see section “Materials and Methods”). Extracts were prepared from liquid cultures shaken for 182 h. We found that deletion of *gcn2* obviously altered the profile of secondary metabolites. New peaks of secondary metabolites that had not been observed before in the WT were detected in *Δgcn2*. For instance, peaks with retention times of 6 and 13 min (**Figure 5A**, second panel from the top). A few minor peaks were observed for NK17, but disappeared in the mutant strain (**Figure 5A**). Interestingly, the polyketide pestalotiollide B that we described before was detected in both the WT and the mutant strain (*Δgcn2*) at a similar level (Niu et al., 2015). Most significantly, when strains were cultured in the presence of 3AT (5 mM) to mimic amino acid starvation conditions, the general production of secondary metabolites in *Δgcn2*, including pestalotiollide B, was almost inhibited (**Figure 5B**). The production of secondary metabolites was restored to the WT level and pattern in the profile of the complementation strain (**Figure 5B**, bottom panel). As we mentioned above, loss of *gcn2* led to a less-pigmented phenotype of the fungus (**Figure 3A**, the Asn panel). The above results demonstrate that *gcn2* is a regulator of secondary metabolism (including melanin production) and may be involved in either activation or silencing of gene clusters in NK17 (the former as deletion of *gcn2* resulted in the formation of new products) (**Figure 5A**).

### Gcn2 Is Critical for the Transcription of Target Genes

**FIGURE 6:**
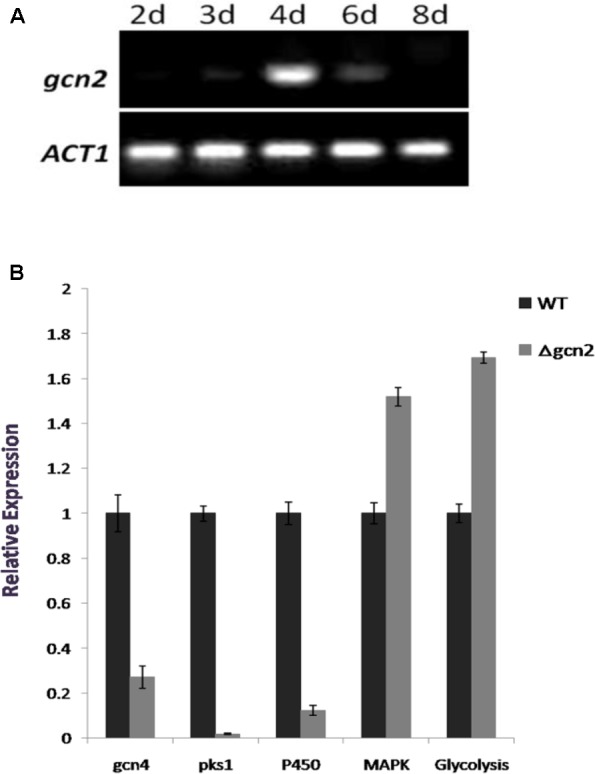
Expression analyses by RT-PCR and real-time qPCR. **(A)** Time-dependent expression of *gcn2* determined by reverse transcription PCR. The expression of *ACT1* (encoding actin) was used as an internal control. **(B)** Quantitative analysis of *gcn2* target gene expression by qRT-PCR. The expression of *gcn4*, *pks1*, and a P450-encoding gene fell significantly in *Δgcn2*, whereas expression of two other genes, a MAPK and a gene involved in glycolysis, increased. These genes were chosen from a RNA-Seq analysis (unpublished data). The RT-PCR was performed in triplicate, and errors are expressed as the standard deviation.

We found that expression of *gcn2* occurred in a time-dependent manner (**Figure 6A**). The transcription of *gcn2* started on the 2nd day of shaking and it reached its maximal expression level on the 4th day. After that, the expression decreased swiftly. In *S. cerevisiae*, in response to the depletion of amino acids, Gcn2 phosphorylates eIF2α, leading to a decrease of global protein synthesis, while activating the expression of a subset of genes such as *gcn4* (Chaveroux et al., 2010). *gcn4* encodes a transcription factor that regulates the expression of myriad target genes. We identified a *gcn4* counterpart in NK17. The qRT-PCR results suggest that the mRNA level of *gcn4* decreased dramatically in strain *Δgcn2*, to only 37% of that in WT NK17 (**Figure 6B**), suggesting that Gcn2 affects the expression of *gcn4* at the mRNA level in *P. microspora*.

We previously demonstrated that a polyketide synthase gene *pks1* was responsible for the pigmentation of the conidia in *P. microspora* (Yu et al., 2015a). The qRT-PCR data (**Figure 6B**) showed a dramatic fall of the mRNA level of *pks1*, and a few other genes, for instance, one of the P450 family genes (GenBank accession no. MF564072). The mRNA level of the P450-encoding gene and *pks1* decreased sharply, by 9.6-fold and 48-fold in *Δgcn2*, respectively, compared with their mRNA levels in the WT (**Figure 6B**). However, a subset of genes was upregulated by the disruption of *gcn2* (identified by a RNA-Seq service; data not shown) (**Figure 6B**). The qRT-PCR data indicates that Gcn2 is a global regulator in *P. microspora*.

## Discussion

In this study, we showed that *P. microspora* strain NK17 has a single eIF2 kinase GCN2 locus that encodes a protein with sequence similarity to GCN2 proteins from other organisms (**Figures 1A,C**). By analogy with *S. cerevisiae* (Castilho et al., 2014), we predicted that Gcn2 might play an important role in the *P. microspora* GCN response. Some of our experimental results confirmed that this is the case. We observed that NK17 *gcn2* was required for the survival of the fungus in amino acid starvation conditions (**Figure 3A** second row of plates, **Figure 3C**), ratifying that this kinase plays a conserved role in amino acid anabolism in *P. microspora*.

We actually found in this study a novel function of *gcn2* regulating the production of the asexual spore conidia. Our data (**Figure 3B**) clearly demonstrate that the function of *gcn2* is critical for the conidiation process in *P. microspora*. Although the molecular mechanisms regulating asexual sporulation are still not fully understood, there has been some progress in understanding the genetic regulation of conidiation and the effects of light (Park and Yu, 2012). However, there is little information about the function of *gcn2* in the development of conidia in filamentous fungi. Given that pigmentation of NK17 is tightly associated with the formation of conidia (Yu et al., 2015a), our data confirmed that loss of melanin is consistent with the deficiency of conidiation. Another interesting finding is that *gcn2* was expressed in a time-dependent manner in *P. microspora*. Its highest expression level was reached on day four (**Figure 6A**). *P. microspora* NK17 usually starts conidiation in the culture after 4 days, supporting the observation that *gcn2* is involved in the process of conidiation (**Figure 3B**). Taking together, the role of *gcn2* in conidiation and melanin production observed in this study suggests a connection between asexual reproduction and amino acid synthesis in *P. microspora*.

Filamentous fungi produce a number of small bioactive molecules as part of their secondary metabolism, and secondary metabolism can be linked to fungal developmental programs in response to various abiotic or biotic external triggers (Bayram and Braus, 2012). Some secondary metabolites are produced by common biosynthetic pathways, often in conjunction with morphological development (Keller et al., 2005), but many secondary metabolites are not well characterized, genetically or structurally (Weber et al., 1994). Most secondary metabolites are derived from either non-ribosomal peptides (NRPs) or polyketides (Brakhage, 2013). In *P. microspora*, pestalotiollide B, a polyketide derivative, is likely synthesized by fungal polyketide synthase (PKS) (Chen et al., 2017). It is structurally related to dibenzodioxocinones and penicillide, which are also natural products of fungi and represent a new class of CETP inhibitors (Bruckner et al., 2005; Niu et al., 2015; Chen et al., 2017). We were interested in finding the possible regulatory role of *gcn2* in the production of secondary metabolites in either normal or amino acid-starved conditions. Our HPLC profiling data for strain *Δgcn2* revealed that *gcn2* participates in the regulation of the biosynthesis of secondary metabolites (**Figure 5A**). At least two new peaks emerged in the HPLC metabolite profile of the mutant strain *Δgcn2*. Therefore, the biosynthesis of these two metabolites is likely negatively regulated by Gcn2. The complementation strain displayed a different profile from the WT, indicating that the expression of the reintroduced copy of *gcn2* was distinct in the complemented strain. This may be because, for example, the insertion locus was changed. The production of pestalotiopsis B (PB) remained almost unchanged in all the strains, suggesting its synthesis is not under the control of *gcn2* (indicated by an arrow, **Figure 5A**). However, on amino acid starvation (in the presence of 3AT), deletion of *gcn2* almost abolished the biosynthesis of secondary metabolites (**Figure 5B**). The above results clearly suggest that *gcn2* can function as a critical global regulator controlling secondary metabolism, in particular in conditions of amino acid paucity. Nonetheless, *cpc1*, the equivalent of *gcn2* in *A. nidulans*, shows disparate roles in secondary metabolite production. *A. nidulans cpc1* negatively regulates the genes for production of penicillin (Braus et al., 2003). Similar was reported for *Leptosphaeria maculans*, in which *cpcA*/*cpc1* had a negative regulatory role on the secondary metabolite sirodesmin PL. Silenced *cpcA/cpc1* resulted in much higher amounts of the product than were observed in the WT (Elliott et al., 2011). In *Fusarium fujikuroi*, *cpc1* was dispensable for secondary metabolism (Schonig et al., 2009). Thus, Gcn2/Cpc1 kinases seem to play divergent roles in the regulation of secondary metabolism in filamentous fungi.

Roles of Gcn2 in stress response other than amino acid starvation have also been reported in yeast, e.g., in glucose starvation and in tolerance to oxidative stress and antifungal agents. We tested the role of *P. microspora* Gcn2 in stress conditions. The growth of strain *Δgcn2* showed slight but discernable differences compared with the WT and the complementation strain under stress (**Figure 4**). It is worth noting here that *P. microspora* Gcn2 was required for tolerance to 0.02% Congo red and 50 ng/mL calcofluor white. The mutant strain apparently grew slower in the dyes than the WT and the complementation strain (**Figure 4**). The main components of cell walls are (1, 3) β-glucans, (1, 6) β-glucans, chitin, and mannose in yeast (Lesage and Bussey, 2006), and the general effects of calcofluor white and Congo red on fungal wall morphogenesis are on the biosyntheses of (1, 3) β-glucans and chitin during cell growth and protoplast wall generation (Roncero and Duran, 1985; Lipke and Ovalle, 1998). Our data demonstrate that Gcn2 is essential to the integrity of the cell wall in *P. microspora* NK17. Additionally, it is reported that the high osmolarity glycerol pathway in fungi is responsible for resistance to osmotic stress, including to high concentrations of KCl or NaCl in *C. neoformans* (Bahn et al., 2005). Sensitivity to 0.5 M KCl and 1 M NaCl observed for strain *Δgcn2* in this study suggests a possible role of Gcn2 in tolerance of this stress.

Activated Gcn2 phosphorylates eIF2α in *S. cerevisiae*, which leads to specific protein synthetic increases, e.g., of Gcn4 (Baird and Wek, 2012). We found that the expression of the *gcn4* counterpart in *P. microspora* fell markedly in *Δgcn2* (**Figure 6B**), suggesting *gcn2* affects the expression of *gcn4* at the transcriptional level. PB is a polyketide derivative; its biosynthesis may require the action of PKSs, which are usually synthesized by type I iterative PKSs that contain multiple enzymatic domains (Brakhage, 2013; Chen et al., 2017). In *P. microspora*, *pks1* encodes a PKS, and the expression of *pks1* that is responsible for conidial melanin production in NK17 and a P450 family gene both required the function of Gcn2 for transcription (**Figure 6B**). However, a MAPK family gene and a glycolysis family gene were upregulated in the absence of *gcn2*. These results suggest that *gcn2* affects multiple pathways in *P. microspora*. In *Neurospora crassa*, Cpc1 controls at least 443 target genes (Tian et al., 2007).

In summary, we demonstrated in this study the importance of the *gcn2* kinase gene as a global regulator in growth in amino acid starvation, biosynthesis of secondary metabolites, conidial development, and cell wall integrity in *P. microspora*. This work may provide information for manipulating metabolic pathways to produce new products in this fungus.

## Author Contributions

XZ and DW conceived and designed the study. DW, OA, XH, and XY performed the experiments. LC provided the mutants. DW and YL wrote the paper. DW and XZ reviewed and edited the manuscript. All authors read and approved the manuscript.

## Conflict of Interest Statement

The authors declare that the research was conducted in the absence of any commercial or financial relationships that could be construed as a potential conflict of interest.
